# Site-specific regulation of histone H1 phosphorylation in pluripotent cell differentiation

**DOI:** 10.1186/s13072-017-0135-3

**Published:** 2017-05-22

**Authors:** Ruiqi Liao, Craig A. Mizzen

**Affiliations:** 1 Department of Cell and Developmental Biology, University of Illinois at Urbana Champaign, B107 Chemistry and Life Sciences Building, MC-123 601 S. Goodwin Ave., Urbana, IL 61801 USA; 2 Institute for Genomic Biology, University of Illinois at Urbana Champaign, Urbana, IL 61801 USA

**Keywords:** Histone H1, Phosphorylation, Embryonic stem cell, Pluripotency factors, Cell differentiation, Cyclin-dependent kinase (CDK), CDK2, CDK7, CDK9, P-TEFb

## Abstract

**Background:**

Structural variation among histone H1 variants confers distinct modes of chromatin binding that are important for differential regulation of chromatin condensation, gene expression and other processes. Changes in the expression and genomic distributions of H1 variants during cell differentiation appear to contribute to phenotypic differences between cell types, but few details are known about the roles of individual H1 variants and the significance of their disparate capacities for phosphorylation. In this study, we investigated the dynamics of interphase phosphorylation at specific sites in individual H1 variants during the differentiation of pluripotent NT2 and mouse embryonic stem cells and characterized the kinases involved in regulating specific H1 variant phosphorylations in NT2 and HeLa cells.

**Results:**

Here, we show that the global levels of phosphorylation at H1.5-Ser18 (pS18-H1.5), H1.2/H1.5-Ser173 (pS173-H1.2/5) and H1.4-Ser187 (pS187-H1.4) are regulated differentially during pluripotent cell differentiation. Enrichment of pS187-H1.4 near the transcription start site of pluripotency factor genes in pluripotent cells is markedly reduced upon differentiation, whereas pS187-H1.4 levels at housekeeping genes are largely unaltered. Selective inhibition of CDK7 or CDK9 rapidly diminishes pS187-H1.4 levels globally and its enrichment at housekeeping genes, and similar responses were observed following depletion of CDK9. These data suggest that H1.4-S187 is a *bona fide* substrate for CDK9, a notion that is further supported by the significant colocalization of CDK9 and pS187-H1.4 to gene promoters in reciprocal re-ChIP analyses. Moreover, treating cells with actinomycin D to inhibit transcription and trigger the release of active CDK9/P-TEFb from 7SK snRNA complexes induces the accumulation of pS187-H1.4 at promoters and gene bodies. Notably, the levels of pS187-H1.4 enrichment after actinomycin D treatment or cell differentiation reflect the extent of CDK9 recruitment at the same loci. Remarkably, the global levels of H1.5-S18 and H1.2/H1.5-S173 phosphorylation are not affected by these transcription inhibitor treatments, and selective inhibition of CDK2 does not affect the global levels of phosphorylation at H1.4-S187 or H1.5-S18.

**Conclusions:**

Our data provide strong evidence that H1 variant interphase phosphorylation is dynamically regulated in a site-specific and gene-specific fashion during pluripotent cell differentiation, and that enrichment of pS187-H1.4 at genes is positively related to their transcription. H1.4-S187 is likely to be a direct target of CDK9 during interphase, suggesting the possibility that this particular phosphorylation may contribute to the release of paused RNA pol II. In contrast, the other H1 variant phosphorylations we investigated appear to be mediated by distinct kinases and further analyses are needed to determine their functional significance.

**Electronic supplementary material:**

The online version of this article (doi:10.1186/s13072-017-0135-3) contains supplementary material, which is available to authorized users.

## Background

The H1 family of linker histones is important chromatin architectural proteins that bind linker DNA and facilitate higher order chromatin folding [1–3]. Human cells differentially express 11 genes encoding non-allelic amino acid sequence variants of H1 [4, 5]. These share a common tripartite structure in which a conserved globular domain (GD) is flanked by a short N-terminal domain (NTD) and a longer C-terminal domain (CTD) [6]. Fluorescence recovery after photobleaching (FRAP) microscopy of H1 variant-GFP fusions suggests that the association of H1 with chromatin is highly dynamic in vivo [7, 8] and that variation in CTD structure between individual H1 variants is major determinants of differences in their chromatin binding affinities and dynamics [9, 10].

Several types of post-translational modifications, including phosphorylation, methylation and acetylation, have been identified in H1 variants, with phosphorylation being particularly abundant [11–13]. Analyses of synchronized cells from multiple organisms suggest that H1 phosphorylation increases progressively during interphase before peaking transiently during mitosis [14–19]. However, these early studies did not identify which sites in individual H1 variants are phosphorylated during interphase and mitosis. More recently, mass spectrometry has enabled the precise identification of H1 phosphorylation sites. Phosphorylation at both cyclin-dependent kinase (CDK) consensus motifs and non-CDK sites has been detected [20–23]. Interphase H1 phosphorylation occurs predominantly, if not exclusively, at serine-containing CDK motifs (SPXZ, X = any amino acid, Z = K or R). Mitotic H1 phosphorylation occurs at these same SPXZ sites together with phosphorylation that occurs exclusively during mitosis at TPXZ CDK motifs and some non-CDK sites [21, 23–28]. S18, S173 and S189 were identified as sites of interphase phosphorylation of H1.5 [21, 24], but the significance of these phosphorylations remains unclear [13].

Our laboratory identified H1.2-S173, H1.4-S172 and H1.4-S187 as the predominant sites of interphase phosphorylation of H1.2 and H1.4 in HeLa S3 cells. Analyses with antisera that are highly specific for H1.4 phosphorylation at S187 (pS187-H1.4) suggest that pS187-H1.4 is enriched at sites of transcription by RNA polymerases I and II [23], but how the levels and chromatin distribution of pS187-H1.4 are regulated is unknown. Similarities in the nuclear and nucleolar staining patterns obtained with our antisera to pS187-H1.4 and pS173-H1.2/5 suggest that enrichment of the latter may also correlate with transcription [23], and this is supported by evidence that staining with pS173-H1.2/5 antisera raised by another laboratory colocalizes extensively with Br-UTP labeling of nascent RNA and to a lesser extent with EdU labeling of replicating chromatin, in HeLa and HEK293 cells [24]. However, other interphase phosphorylations may be functionally distinct since staining for pS18-H1.5 showed little colocalization with either Br-UTP or EdU labeling [24]. The data available currently suggest that the impact of interphase S18 phosphorylation in the H1.5 NTD differs from that of the transcription-associated roles proposed for interphase phosphorylations in the CTDs of H1.2, H1.4 and H15. This suggests, in turn, that different kinases may mediate interphase phosphorylation at different sites in H1 variants. Although CDK2 and CDK9 have been implicated as possible interphase H1 kinases [29–33], whether they display site-specificity among H1 variants in vivo is not known.

Recent evidence suggests that individual H1 variants play significant roles in embryonic stem (ES) cell differentiation. Analyses of the abundance of the mRNAs encoding seven somatic H1 variants suggest that they are differentially expressed during the differentiation of human ES cells (hESCs) in vitro [34]. H1 triple-knockout mouse ES cells (mESCs) depleted of H1c, H1d and H1e (H1.2-4) display global alterations in chromatin structure and an impaired capacity for differentiation compared to wild-type mESCs [35, 36]. Comparison of the genomic distribution of H1.5 in hESCs versus differentiated cell lines suggests that H1.5 becomes enriched at specific gene family clusters during cellular differentiation [37]. Although the mechanisms involved have yet to be defined, these findings support an emerging view that individual H1 variants differ in their impact on cellular differentiation. Metazoan H1 variants differ conspicuously from each other in possessing different numbers of sites for interphase phosphorylation that vary in their relative location and amino acid sequence context. Thus, differences in the expression, genomic distribution and interphase phosphorylation dynamics between individual H1 variants may enable them to play distinct roles during ES cell differentiation. However, data on H1 variant phosphorylation dynamics during cell differentiation has not been reported. Here, we show that the global levels of interphase phosphorylation at H1.5-S18 and H1.2/H1.5-S173 decrease to different extents when pluripotent human NTERA-2/D1 (NT2) cells differentiate. Similar decreases in the global levels of pS18-H1.5 and pS173-H1.2/5 occur when mESCs differentiate, but pS173-H1.2/5 levels are reduced more extensively than in NT2 cells. The global level of pS187-H1.4 also decreased when NT2 cells differentiated, but pS187-H1.4 levels at the transcription start sites (TSSs) of pluripotency factor genes fell to a disproportionately greater extent that correlated with their diminished expression. In contrast, the association of pS18-H1.5 and pS173-H1.2/5 with pluripotency factor gene TSSs in differentiated cells tended to increase or decrease, respectively, but these changes did not reach statistical significance. pS18-H1.5, pS173-H1.2/5 and pS187-H1.4 were associated with different extents with housekeeping gene TSSs in undifferentiated cells, and this did not change significantly upon cell differentiation. Contrary to evidence suggesting that CDK2 is involved in interphase H1 phosphorylation, selective inhibition of CDK7 or CDK9, but not CDK2, significantly decreased the global levels of pS187-H1.4, and inhibition of CDK7 or CDK9 rapidly diminished the association of pS187-H1.4 with specific genes in HeLa cells. The likelihood that CDK9 mediates interphase H1.4-S187 phosphorylation was further supported by their colocalization at gene promoters in both HeLa and NT2 cells in reciprocal re-ChIP analyses and by manipulation of the levels of chromatin-associated CDK9. Depletion of CDK9 with siRNA significantly decreased the global level of pS187-H1.4 in HeLa cells, and the level of pS187-H1.4 at housekeeping genes reflected the extent to which CDK9 association was reduced at these sites. In contrast, actinomycin D rapidly induced the enrichment of both CDK9 and pS187-H1.4 at housekeeping genes. Remarkably, none of the inhibitor treatments affected the global levels of pS18-H1.5 or pS173-H1.2/5. Taken together, our data suggest that CDK9 is the predominant interphase kinase for H1.4-S187, but not for H1.5-S18 or H1.2/H1.5-S173 in both HeLa and NT2 cells, and that interphase phosphorylation of H1 variants is regulated in a site-specific and gene-specific fashion that may confer specialized roles to individual H1 variants in chromatin processes.

## Methods

### Cell culture and differentiation

NTERA-2/D1 (NT2) human embryonal testicular teratocarcinoma cells and W4/129S6 mESCs were obtained from Dr. Fei Wang (UIUC). NT2 cells were grown in DMEM + 10% FBS and subcultured by scraping. Differentiation was induced by dissociating cells with trypsin, followed by seeding at a density of 1 × 10^6^ cells per T-75 flask or 1.33 × 10^4^ cells/cm^2^ in DMEM, supplemented with 10% FBS and 10 μM all-trans retinoic acid (RA). mESCs were maintained in DMEM with high glucose, supplemented with 15% FBS (ES-Cult FBS, Stemcell Technologies, 06952), 0.1 mM non-essential amino acids (Gibco), 1 mM sodium pyruvate (Gibco), 0.1 mM β-mercaptoethanol (Sigma), 2 mM l-glutamine (GlutaMAX, Gibco), 1000 U/mL LIF (Nacalai USA, NU0012) and 1× penicillin/streptomycin (Gibco) on gelatin-coated plates. The cells were fed with fresh medium daily and subcultured in new gelatin-coated plates by trypsinization every other day. Differentiation was induced by seeding cells in ES cell medium without LIF. HeLa cells were grown in DMEM + 10% FBS and subcultured by trypsinization. Cells were treated with flavopiridol (NIH AIDS Reagent Program), NU-6140 (Tocris), actinomycin D (Fisher), α-amanitin (Cayman), triptolide (Tocris) or THZ1 (ApexBio) dissolved in DMSO to selectively inhibit RNA Pol II or CDK activities as described in the figure legends.

### Histone preparation and chromatography

Crude histones were extracted from isolated nuclei with 0.4 N H_2_SO_4_ as described previously [38]. Crude H1 was prepared by 5% perchloric acid fractionation of crude histones and recovered by precipitation with 20% (w/v, final concentration) trichloroacetic acid (TCA). Hydrophobic interaction chromatography was performed using a 4.6 mm ID × 100 mm PolyPROPYL A column (PolyLC Inc.) and a multistep linear gradient from buffer A [2.5 M (NH_4_)_2_SO_4_ in 50 mM sodium phosphate, pH 7.0] to buffer B [1.0 M (NH_4_)_2_SO_4_ in 50 mM sodium phosphate, pH 7.0]. Fractions were collected by time, and proteins were recovered by precipitation with 20% TCA.

### siRNA transfection

siRNA transfection was performed using Lipofectamine RNAiMAX (Invitrogen) according to manufacturer’s protocol. Cells were seeded the day before transfection in order to reach 60–80% confluency at the time of transfection. Transfection reagent and siRNA were diluted in Opti-MEM (Gibco), mixed, and incubated for 5 min at room temperature. The complexes were then added directly to cell cultures, and the cells were harvested 72 h later.

### Immunoblotting

Whole-cell lysates or histone extracts were electrophoresed in 15% polyacrylamide gels (6% gel for Pol II blots) containing SDS, transferred to a PVDF membrane and blocked with 5% milk powder in TBS for 1 h at room temperature. The blocked membrane was then incubated with primary antibody at 4 °C overnight, washed with TBST, and incubated with secondary antibody conjugated with HRP (Amersham, NA-931 or NA-934) for 1 h at room temperature, washed with TBST again, developed with chemiluminescence reagents (Thermo, SuperSignal West Pico Chemiluminescent Substrate) and images recorded with a series of lengthening exposures on X-ray films. Bitmap images were generated from selected films using a flatbed scanner and densitometry performed using ImageJ https://imagej.nih.gov/ij/.

The pS18-H1.5 antisera were generated by immunizing rabbits with a synthetic phosphopeptide (CPVEK-phosphoserine-PAKK) conjugated to maleimide-activated keyhole limpet hemocyanin (Thermo Fisher Scientific) using standard procedures. Pan antisera to H1.0 and H1.5 were generated by immunizing rabbits with full-length recombinant human H1.0 or H1.5 as described previously [23]. The antisera to pS187-H1.4, pS173-H1.2/5 and pan-H1.4 (UI-100) have been described previously [23]. The antisera against other histones and pluripotency markers were obtained from Abcam: H1.2 (ab4086), H1.5 (ab24175), histone H3 (ab1791), Oct4 (ab19857), Sox2 (ab97959), Nanog (ab21624). The commercial antibody against pS18-H1.5 (61107) was purchased from Active Motif. The commercial antisera against H1.0 (sc-56695), Pol II (sc-899) and CDK9 (sc-13130, sc-484) were purchased from Santa Cruz Biotechnology, Inc. The antibodies against phosphorylated CTD of RNA Pol II were purchased from Abcam: Pol II pS2 (ab5095), Pol II pS5 (ab5131).

For peptide competition assays, 10 µg of non-phosphorylated (CPVEKSPAKK) or phosphorylated (CPVEKpSPAKK) H1.5-S18 peptides (Genscript) was incubated with 2 µL of primary antisera in a small volume (500 μL) of PBS for 2 h at room temperature. These mixtures were then further diluted in TBST for use in immunoblotting.

### ChIP and re-ChIP

Chromatin immunoprecipitation (ChIP) experiments were performed as described previously with minor modifications [23]. Cells were cross-linked by adding formaldehyde directly to cultures (1% final) and incubating for 8 min at room temperature. 125 mM final glycine was added, and cultures were incubated for 10 min at room temperature. Cells were then washed twice with cold PBS, scraped, and resuspended in ChIP lysis buffer with protease and phosphatase inhibitors. Chromatin was sheared to ~1 kb mean length by repeated cycles of sonication in a 4 °C water bath using a Bioruptor (Diagenode). After centrifuging at 18,000×*g* for 10 min, the supernatants were diluted tenfold with ChIP dilution buffer. Aliquots representing 1–2 × 10^6^ cells in 1.0 ml final volume were used for each pull down. Samples were incubated with specific antibodies [15 μL pS187-H1.4, 30 μL pS173-H1.2/5, 10 μL pS18-H1.5 (Active Motif) or 20 µL CDK9 (Santa Cruz)] at 4 °C overnight. Immunocomplexes were incubated with 50 μL BSA-blocked protein G Dynabeads (Invitrogen) for 4 h at 4 °C, collected using a magnetic rack, and washed sequentially with ChIP wash buffer I, II, III and twice with TE. Beads were eluted twice with 200 μL 1% SDS in 0.1 M NaHCO_3_ at 65 °C for 10 min. The combined eluates were made 200 mM NaCl (final), incubated at 65 °C overnight to reverse cross-links, digested with 50 μg/ml RNase A at 37 °C for 30 min, and then digested with 50 μg/ml proteinase K at 50 °C for 1 h. The DNA fragments were then purified by phenol/chloroform extraction, recovered by ethanol precipitation using 20 μg glycogen as a carrier, and dissolved in 50 μL of deionized water. For re-ChIP assays, immunoprecipitations from the first ChIP were washed sequentially as described above. The immunocomplexes were eluted with 10 mM DTT in TE at 37 °C for 30 min, diluted 20 times with ChIP dilution buffer and then immunoprecipitated with the second antibody using standard ChIP protocol. ChIP products were quantitated by real-time PCR using SYBR Green master mix (Applied Biosystems) and the primers listed in Additional file 1: Table S1.

## Results

### Site-specific changes in global H1 phosphorylation during cell differentiation

We have generated a collection of highly specific antisera, raised against synthetic phosphopeptides, which recognize phosphorylation at single sites that are unique to individual human H1 variants or are shared between just two variants. We have also raised “pan” antisera against individual full-length recombinant human H1 variants that specifically recognize the intended variant regardless of whether it is phosphorylated or not. The former provide a relative measure of the levels of phosphorylation at defined sites between samples, whereas the latter can be used to confirm that equivalent amounts of a particular H1 variant, regardless of phosphorylation status, are present in the samples being compared. The specificity of our antisera to pS173-H1.2/5, pS187-H1.4 and pan-H1.4 has been described previously [23]. The specificity of our antisera to pan-H1.0, pan-H1.5 and pS18-H1.5 is shown in Additional file 2: Figure S1. We used these antisera and commercially available reagents in immunoblotting to monitor the relative expression and phosphorylation of H1 variants in NT2 cells during seven days of retinoic acid (RA)-induced differentiation. RA induces pluripotent NT2 cells to differentiate along a neural lineage [39, 40]. For comparison, we also analyzed the spontaneous differentiation of pluripotent mouse embryonic stem cells (mESCs) after the removal of leukemia inhibitory factor (LIF) [41, 42]. The sequences of the phosphopeptide antigens used to generate the pS173-H1.2/5, pS187-H1.4 and pS18-H1.5 antisera are completely conserved in the respective mouse H1 variants, and these antisera display the same apparent affinity and specificity for the respective phosphorylated H1 variants in analyses of murine and human samples. Our antisera raised against recombinant human H1 variants are specific for the corresponding mouse H1 variant, but some bind the mouse protein with lower apparent affinity compared to the human (see below).

**Fig. 1:**
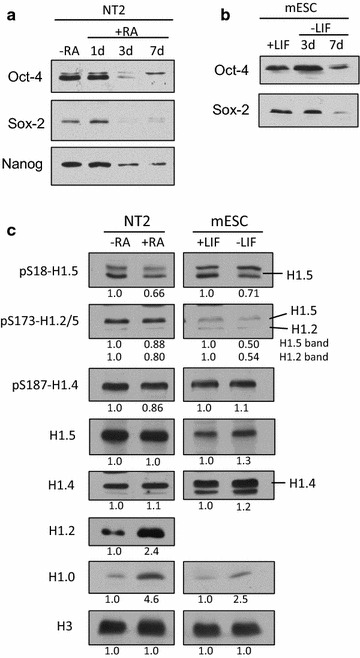
H1 variant phosphorylation is altered at specific sites after pluripotent cells differentiate. **a** Retinoic acid-induced differentiation of NT2 cells was assessed by immunoblotting whole-cell lysates with antibodies specific for the indicated pluripotency factors. **b** Differentiation of mouse embryonic stem cells after withdrawal of leukemia inhibitory factor (LIF) was assessed by immunoblotting whole-cell lysates with antibodies specific for the indicated pluripotency factors. **c** H1 variant expression and the global levels of their phosphorylation at specific sites in NT2 and mouse ES cells before (day 0) and after differentiation (day 7 of RA treatment or LIF withdrawal) were assayed by immunoblotting whole-cell lysates with the indicated antisera. Signals for histone H3 demonstrate equivalent loading for the NT2 and mESC samples, respectively. The *numbers below each panel* indicate densitometry for the day 7 sample relative to the day 0 sample

Control analyses revealed that expression of the Oct-4, Sox-2 and Nanog transcription factors all decreased significantly in NT2 cells after RA addition, confirming differentiation and the loss of pluripotency in cultures during the treatment interval (Fig. 1a). Similarly, Oct-4 and Sox-2 expression in mESCs dropped markedly within 1 week after LIF was removed (Fig. 1b). Comparing the expression and phosphorylation of individual H1 variants relative to histone H3 expression in NT2 cells on days 0 and 7 of RA treatment revealed changes in the global levels of phosphorylation at some sites that did not correlate with changes in H1 variant expression and were presumably related to changes in H1 phosphorylation regulation during the transition to a longer cell cycle in differentiated cells [43]. The level of pS18-H1.5 was markedly lower, and pS173-H1.5 was reduced to a lesser extent, in differentiated NT2 cells even though H1.5 expression was not altered (Fig. 1c). In contrast, the levels of pS187-H1.4 and pS173-H1.2 were reduced in differentiated NT2 cells even though H1.2 and H1.4 expression was increased. Notably, the data suggest that the global levels of phosphorylation at each of these four sites changed in a site-specific fashion. Expression of H1.0 was also upregulated in differentiated NT2 cells, consistent with previous evidence that H1.0 expression is induced during differentiation of NT2 and human ES cells [34]. Although these authors did not detect changes in the relative levels of other H1 variant proteins despite significant changes in mRNA levels, our data suggest that expression of H1.2 and H1.4 also increases when NT2 cells differentiate.

We used hydrophobic interaction chromatography (HIC) as an antibody-independent approach to analyze crude H1 from pluripotent and differentiated NT2 cells. HIC is a convenient alternative to two-dimensional approaches for monitoring H1 variant modification dynamics that provides partial or complete resolution of H1 variants and their phosphorylated forms [23]. HIC analyses revealed that pluripotent NT2 cells express H1.2, H1.3, H1.4 and H1.5 predominantly, and that higher levels of phosphorylation affect H1.5 compared to the other variants. H1.4 and H1.2 were phosphorylated to lesser extents, while phosphorylation of H1.3 was not readily detectable (Additional file 3: Figure S2). Following the addition of RA, the level of H1.5 monophosphorylation (H1.5-1p) appeared to remain stable for the first three days but then decreased markedly between days 3 and 7 of RA treatment. Previous analyses of H1 variant phosphorylation in several cell lines suggest that H1.5-1p is comprised of a mixture of molecules monophosphorylated at S18 (pS18-H1.5) or S173 (pS173-H1.5) [21, 24]. Thus, the decrease in the H1.5-1p peak in HIC is consistent with the decreased signals observed for pS18-H1.5 and pS173-H1.5 in immunoblotting (Fig. 1c). RA treatment also led to diminished H1.4 monophosphorylation (H1.4-1p), but the kinetics differed from those of H1.5-1p. Loss of H1.4-1p was apparent after one day of RA treatment and appeared to decrease somewhat further over the following 6 days. Since monophosphorylation of H1.4 occurs exclusively or predominantly at S187 (pS187-H1.4) [23], the decrease observed for the H1.4-1p peak in HIC is consistent with the reduction in signal observed for pS187-H1.4 in immunoblotting. In contrast, the level of H1.2 monophosphorylation appeared to remain constant throughout the treatment interval (Additional file 3: Figure S2), while the peak for non-phosphorylated H1.2 seemed to increase. Therefore, the ratio of H1.2-1p relative to H1.2-0p was reduced after 7d of RA-induced differentiation, which is consistent with the decrease of pS173-H1.2 detected on blots. Together, the HIC and immunoblotting results suggest that the relative levels of global interphase phosphorylation at pS18-H1.5, pS173-H1.2, pS173-H1.5 and pS187-H1.4 decrease in a site-specific fashion during the differentiation of pluripotent NT2 cells.

Some, but not all, of these changes were observed when mES cells differentiated. In particular, the level of pS18-H1.5 in differentiated mESC was also markedly decreased (Fig. 1c). However, greater reductions in the phosphorylation of both H1.2-S173 and H1.5-S173 were apparent when mESC differentiated compared to NT2 cells. In contrast to NT2 cells, differentiation led to an increase in the level of pS187-H1.4 in mESC, providing further evidence that interphase H1 phosphorylation is regulated in a cell type-specific and site-specific fashion. Moreover, whereas the expression of H1.0 and H1.2 increased with differentiation in NT2 cells, the expression of H1.0, H1.4 and H1.5 increased upon mESC differentiation (Fig. 1c). We were unable to monitor H1.2 expression in mESC because the commercial antisera we used does not detect murine H1.2. Although stronger signals were obtained for total H1.5 in NT2 cells compared to mESCs, this appears to reflect weaker binding of mouse H1.5 by our pan-H1.5 antiserum due to species-specific amino acid sequence differences rather than a marked difference in abundance. Stained gels suggest that H1.5 is present at similar levels in both cell types (data not shown). Our findings that the expression of H1.0, H1.4 and H1.5 increases upon LIF withdrawal is partially consistent with a previous report that the expression of H1.0, H1.2, H1.3 and H1.4 increased when a different mESC line was differentiated to form embryoid bodies [36]. Taken together, the data presented here and findings from previous reports suggest that enhanced H1.0 expression and diminished interphase phosphorylation of H1.5-S18 and H1.5-S173 are common features of pluripotent cell differentiation. Additional changes in H1 variant expression and phosphorylation may also occur depending on the cell type and the differentiation pathway.

### Enrichment of pS187-H1.4 at promoters is associated with pluripotency factor gene transcription

**Fig. 2:**
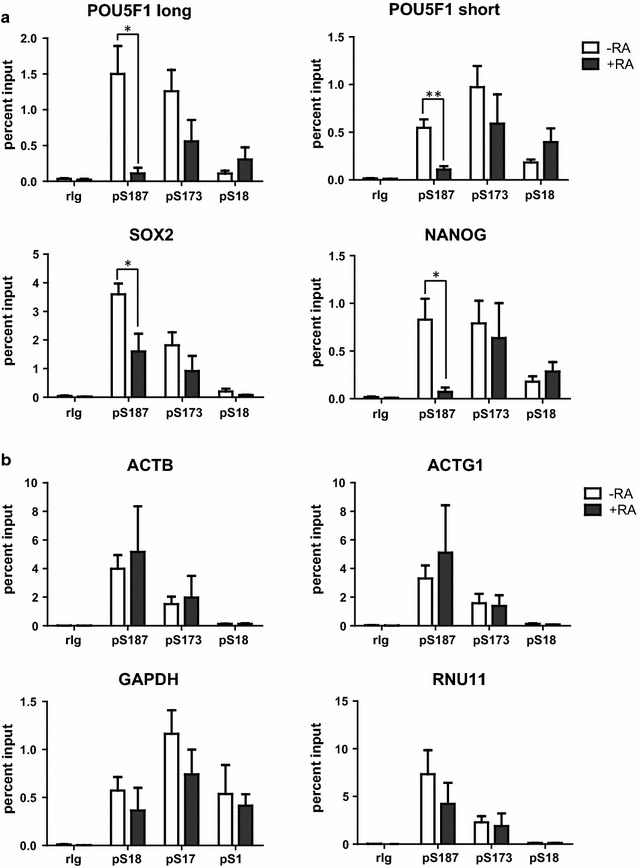
Changes in the levels of phosphorylated H1 variants at pluripotency factor gene promoters correlate with their reduced expression in differentiated NT2 cells. **a** The levels of pS187-H1.4, pS173-H1.2/5 and pS18-H1.5 at the transcription start sites of pluripotency factor genes in NT2 cells before and after 7 days of RA treatment were assessed by ChIP-qPCR. **b** The levels of pS187-H1.4, pS173-H1.2/5 and pS18-H1.5 at the transcription start sites of housekeeping genes in NT2 cells before and after 7 days of RA treatment were assessed by ChIP-qPCR. Negative control ChIP assays employed non-immune rabbit immunoglobulin (rIg) in place of primary antisera. Custom antisera were used for pS187-H1.4 and pS173-H1.2/5 ChIP. Commercial antisera (Active Motif) was used for pS18-H1.5 ChIP. The data are expressed as the percent relative to input DNA (mean ± s.e.m., *p < 0.05; **p < 0.01)

Since the global levels of pS18-H1.5, pS173-H1.2/5 and pS187-H1.4 appeared to decrease by different extents contemporaneously with the downregulation of pluripotency factor expression when NT2 cells differentiated, we used ChIP-qPCR to investigate whether changes in the association of these phosphorylated forms at pluripotency genes might contribute to their transcriptional regulation. As shown in Additional file 4: Figure S3, pS187-H1.4 is enriched at the promoters of the active ACTG1 and GAPDH genes in HeLa cells, but not at the promoter of the repressed MYOD1 gene or an intergenic region within the chromosome 1p35.3 band, consistent with our prior evidence that enrichment of pS187-H1.4 may serve as a general marker for transcriptionally active chromatin [23]. pS187-H1.4 was enriched at the transcription start site (TSS) of pluripotency genes (Fig. 2a) and housekeeping genes (Fig. 2b) in pluripotent NT2 cells. Upon differentiation, the level of pS187-H1.4 at the TSSs of pluripotency factor genes fell markedly, whereas the levels at housekeeping gene TSSs either increased or decreased, but these changes were not statistically significant. Although the levels of pS187-H1.4 decrease globally during NT2 cell differentiation (Fig. 1; Additional file 3: Figure S2), the latter finding implies that pS187-H1.4 is selectively diminished at pluripotency factor gene promoters during NT2 cell differentiation. This notion is further supported by the fact that the magnitude of the loss of pS187-H1.4 at all four pluripotency factor promoters was greater than the loss detected at the global level. Similarly, we found that pS173-H1.2/5 association with the TSSs of pluripotency genes, but not housekeeping genes, decreased after differentiation, although the changes were not statistically significant (Fig. 2a). Since the pS173-H1.2/5 antibody detects phosphorylation on both H1.2 and H1.5, it is unclear whether the decrease at pluripotency gene TSSs in differentiated NT2 cells is due to changes in the association of both pS173-H1.2 and pS173-H1.5 or the selective loss of one form over the other. However, since the global level of pS173-H1.2 was much lower than that of pS173-H1.5 in both pluripotent and differentiated NT2 cells (Fig. 1; Additional file 3: Figure S2), it is possible that the decrease in ChIP signal reflects selective depletion of pS173-H1.5 from pluripotency gene promoters in differentiated cells.

Although the decrease in the global level of pS18-H1.5 in differentiated NT2 cells was notable (Fig. 1; Additional file 3: Figure S2), the level of pS18-H1.5 at the promoters we assessed did not change significantly (Fig. 2a, b). This finding is consistent with prior evidence that pS18-H1.5 is not enriched in active chromatin [24]. Taken together, the results in Fig. 2 suggest that enrichment of pS187-H1.4, and possibly pS173-H1.2 and pS173-H1.5, at gene promoters correlates with their transcription, whereas dephosphorylation or depletion of these forms correlates with their repression. Our results also suggest that the association of interphase phosphorylation at the H1.5-S18 NTD site with transcription may differ, in general, from that of the H1.2/H1.5-S173 and H1.4-S187 CTD sites.

### Interphase phosphorylation of H1.4-S187 and H1.5-S18 are regulated disparately

**Fig. 3:**
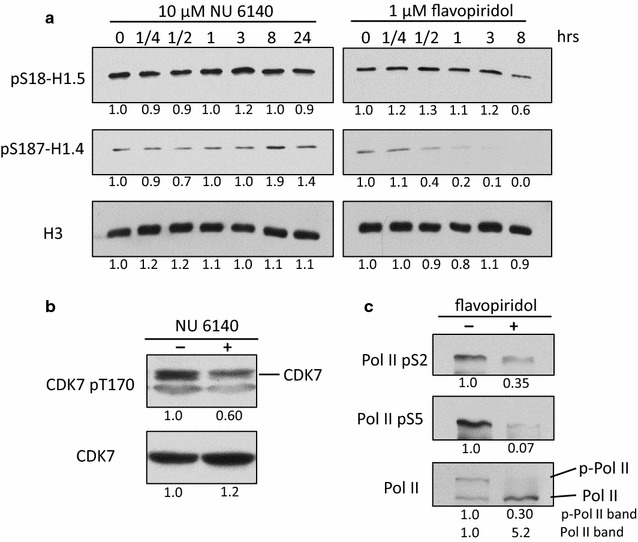
The activity of CDK9, but not CDK2, is required for H1.4-S187 phosphorylation. **a** Pluripotent NT2 cells were treated for increasing intervals with 10 µM NU6140 or 1 µM flavopiridol to preferentially inhibit CDK2 and CDK9, respectively. The global abundance of pS18-H1.5 and pS187-H1.4 was assessed by immunoblotting whole-cell lysates. The blot for histone H3 serves as a loading control. The 0 h time points provide a solvent control (DMSO). Commercial antisera (Active Motif) was used to detect pS18-H1.5. **b** HeLa cells were treated with DMSO or 10 μM NU6140 for 1 h. The loss of CDK2-mediated phosphorylation of CDK7-T170 [50] was assessed by immunoblotting whole-cell lysates with the indicated antisera. **c** HeLa cells were treated with DMSO or 1 μM flavopiridol for 1 h. Altered phosphorylation in the CTD heptad repeats of RNAP II [51] was assessed by immunoblotting whole-cell lysates with the indicated antisera. The *numbers below each panel* indicate densitometry for the treated samples relative to the respective control sample

The evidence that phosphorylation at the H1.5-S18 NTD site may impact transcription differently than phosphorylation at the H1.4-S187 CTD site prompted us to investigate how these phosphorylations are regulated. Both CDK2 and CDK9 have been implicated previously in interphase H1 phosphorylation, but the evidence linking either kinase to the phosphorylation of specific sites in individual H1 variants in vivo is ambiguous [29–33]. Consequently, we assessed the global levels of pS18-H1.5 and pS187-H1.4 in pluripotent NT2 cells after selective inhibition of CDK2 with NU6140 or CDK9 with flavopiridol (FLVP) [44–46]. Much of the CDK9 present in cells is associated with cyclin T in the P-TEFb complex that phosphorylates the CTD of RNAP II, DRB sensitivity inducing factor (DSIF), and negative elongation factor (NELF) to release promoter-proximal paused RNAP II and activate transcriptional elongation [47, 48]. Remarkably, the levels of pS18-H1.5 were unaffected during 24 h of NU6140 treatment (Fig. 3a). pS18-H1.5 levels were also stable during the initial 3 h of FLVP treatment, but a significant decrease was apparent after 8 h of treatment. Since the delayed onset of diminished pS18-H1.5 may be attributable to inhibition of cell cycle progression by FLVP [49], our data suggest it is unlikely that either CDK2 or CDK9 is involved in regulating H1.5-S18 interphase phosphorylation. Control experiments confirmed that our preparations of NU6140 and FLVP were active and attenuated the phosphorylation of CDK7 and the RNAP II-CTD, known targets of CDK2 and CDK9, respectively (Fig. 3b, c) [50, 51]. Similarly, pS187-H1.4 levels remained unchanged during the first 3 h of NU6140 treatment, but were increased after 8 and 24 h of treatment, suggesting that CDK2 also does not directly regulate H1.4-S187 interphase phosphorylation. In contrast, FLVP treatments as short as 30 min markedly reduced the global levels of pS187-H1.4, suggesting that H1.4-S187 is a *bona fide* CDK9 substrate or that CDK9 is otherwise involved in regulating the global levels of this modification.

### Inhibiting CDK7 or CDK9 rapidly diminishes global and gene-specific levels of pS187-H1.4

**Fig. 4:**
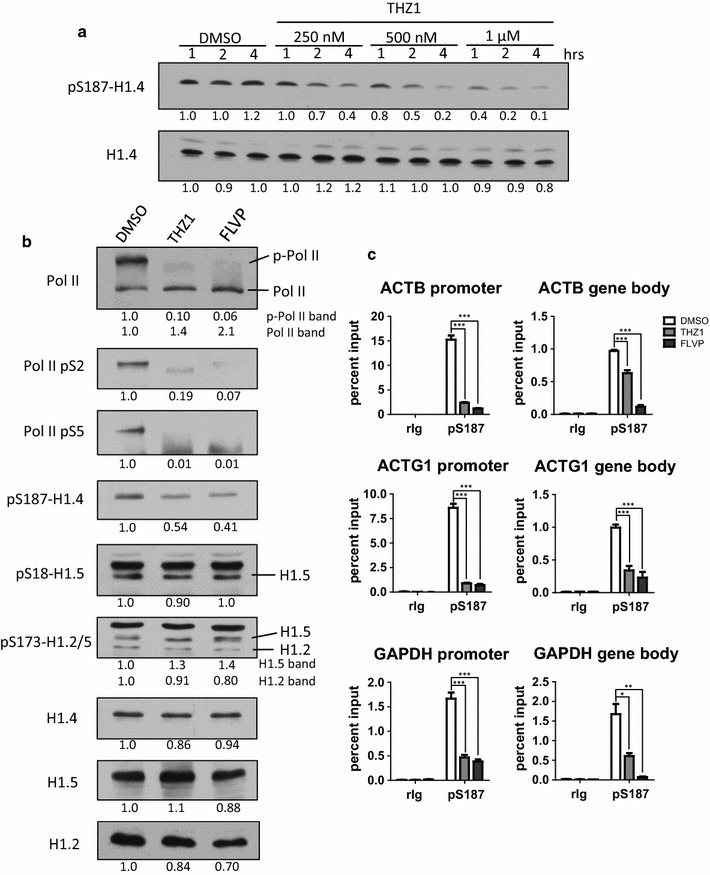
Selective inhibition of CDK7 or CDK9 diminishes pS187-H1.4 levels globally and at specific genes. **a** HeLa cells were treated with DMSO (solvent control) or increasing amounts of THZ1 to selectively inhibit CDK7 for 1, 2 or 4 h. The global abundance of pS187-H1.4 and H1.4 was assessed by immunoblotting whole-cell lysates. **b** HeLa cells were treated with DMSO (solvent control), 1 μM THZ1 or 1 μM FLVP for 1 h to selectively inhibit CDK7 and CDK9, respectively. Immunoblotting of whole-cell lysates with the indicated antisera was used to monitor the abundance of phosphorylated forms of RNAP II and selected H1 variants. The *numbers below each panel* indicate densitometry for the treated samples relative to the control sample. **c** HeLa cells were treated as in (**b**) and the levels of pS187-H1.4 at the promoters or gene bodies of housekeeping genes were assessed by ChIP-qPCR. Negative control ChIP assays employed non-immune rabbit immunoglobulin (rIg) in place of primary antisera. The data are expressed as percent relative to input DNA (mean ± s.e.m., *p < 0.05; **p < 0.01; ***p < 0.001)

Although FLVP inhibits CDK9 preferentially [45], inhibition of additional kinases by FLVP [52–54] could reduce pS187-H1.4 levels and bias the identification of CDK9 as a major kinase for H1.4-S187. We investigated this possibility using THZ1, a recently developed inhibitor that exhibits extraordinary selectivity for CDK7 due to a novel mechanism of inhibition that involves covalent binding to a cysteine residue outside of the kinase domain that is unique to CDK7 [55]. As a subunit of both the CDK-activating kinase (CAK) and TFIIH multi-protein complexes, CDK7 is involved in regulating cell cycle progression and transcription [56]. TFIIH affects transcription via several mechanisms, including phosphorylating the heptapeptide repeats of the RNAP II-CTD at Ser5 and Ser7 during initiation and promoter clearance, and phosphorylating Thr186 in the T-loop of CDK9 to activate phosphorylation of RNAP II-CTD heptads at Ser2 and the release of paused RNAP II by the P-TEFb complex [57–59]. We reasoned that if H1.4-S187 was a *bona fide* CDK9 substrate, selective inhibition of CDK7 by THZ1 should impair CDK9 (P-TEFb) activation and lead to decreased levels of pS187-H1.4. Initial experiments revealed that THZ1 reduced global levels of pS187-H1.4 in HeLa cells in a dose- and time-dependent manner (Fig. 4a). We then compared the effects of treating HeLa cells for just one hour with 1 µM THZ1 or 1 µM FLVP. Both drugs significantly decreased the phosphorylation of the RNAP II-CTD and markedly suppressed global pS187-H1.4 levels, with FLVP eliciting a greater reduction than THZ1 (Fig. 4b). Remarkably, the level of pS18-H1.5 was not affected by either treatment. Both THZ1 and FLVP led to slight reductions in the level of pS173-H1.2, but these may have been attributable to reduced expression of H1.2. Both treatments also led to increases in the level of pS173-H1.5 that did not appear to be attributable to increased H1.5 expression. These data provide additional evidence that different kinases are involved in regulating interphase phosphorylation of H1.4-S187 versus H1.5-S18, H1.2-S173 and H1.5-S173, but differences in the rates of phosphoryl turnover at these sites could also be involved.

One hour treatments with THZ1 or FLVP markedly decreased the levels of pS187-H1.4 at the promoters and bodies of three housekeeping genes (Fig. 4c). However, as we found for global pS187-H1.4 levels, the decreases observed for FLVP were larger than those for THZ1. Moreover, the effect of THZ1 was more pronounced at these promoters compared to the gene bodies. Given the evidence that CDK9 activity is regulated by CDK7 [55, 59], our data support models in which CDK7 indirectly regulates pS187-H1.4 levels at promoters by controlling CDK9 (P-TEFb) activation, whereas CDK9 (P-TEFb) or other FLVP-sensitive kinases associated with elongating RNAP II mediate H1.4-S187 phosphorylation in gene bodies [60]. However, our data do not exclude the possibility that H1.4-S187 may also be a *bona fide* substrate for CDK7.

### Depletion of CDK9 decreases the global and gene-specific levels of pS187-H1.4

**Fig. 5:**
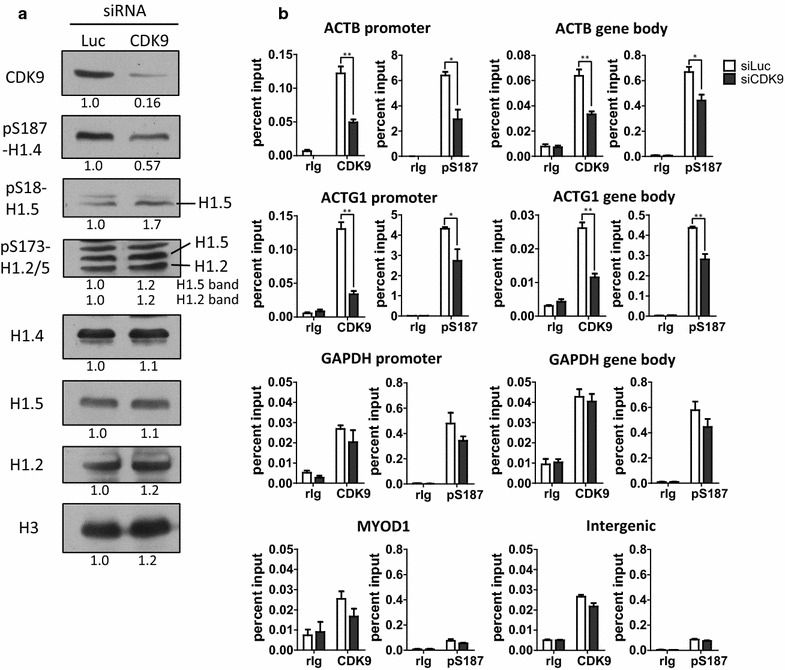
siRNA depletion of CDK9 reduced the global and gene-specific levels of pS187-H1.4. **a** HeLa cells were transfected with siRNA against CDK9 or control siRNA against luciferase for 72 h. Immunoblotting of whole-cell lysates with the indicated antisera was used to assess CDK9 expression and the abundance and phosphorylation of selected H1 variants. The *numbers below each panel* indicate densitometry for the CDK9-depleted sample relative to the luciferase siRNA control sample. **b** HeLa cells were transfected with siRNAs against luciferase or CDK9 and the levels of CDK9 and pS187-H1.4 at the promoters or gene bodies of housekeeping genes were assessed by ChIP-qPCR. Negative control ChIP assays employed non-immune rabbit immunoglobulin (rIg) in place of primary antisera. The data are expressed as the percent relative to input DNA (mean ± s.e.m., *p < 0.05; **p < 0.01)

To confirm that the effects observed for THZ1 or FLVP treatment were due to inhibition of CDK9, we assessed how depletion of CDK9 affected pS187-H1.4 levels in HeLa cells. Despite overloading of the CDK9-depleted sample for the repeat shown in Fig. 5 (compare pan-H3 levels), immunoblots revealed that very little CDK9 perdured after 72 h of siRNA treatment, and this was associated with a marked reduction in the global level of pS187-H1.4 (Fig. 5a). In contrast, the global levels of pS18-H1.5, pS173-H1.2 and pS173-H1.5 were all increased in the CDK9-depleted sample, but this may reflect the enhanced loading of this sample as noted above. These data, together with the results from the kinase inhibitor treatments (Figs. 3, 4), strongly suggest that interphase H1.4-S187 phosphorylation depends on the activity of CDK9, whereas other kinases mediate interphase phosphorylation of H1.5-S18, H1.2-S173 and H1.5-S173.

CDK9 depletion led to striking reductions in the association of both CDK9 and pS187-H1.4 with the promoters and bodies of the ACTB and ACTG1 genes that were not observed in the MYOD1 promoter and intergenic negative control regions (Fig. 5b). However, CDK9 depletion did not have significant effects on the binding of CDK9 and pS187-H1.4 at the GAPDH promoter or gene body. These data provide further evidence that CDK9 phosphorylates H1.4-S187, and that the level of pS187-H1.4 at genes is directly related to the extent of co-enrichment of CDK9. They also suggest that the extent of CDK9 dynamics at different genes may vary considerably.

### Phosphorylation at S187-H1.4 is not dependent on RNAP II progression

**Fig. 6:**
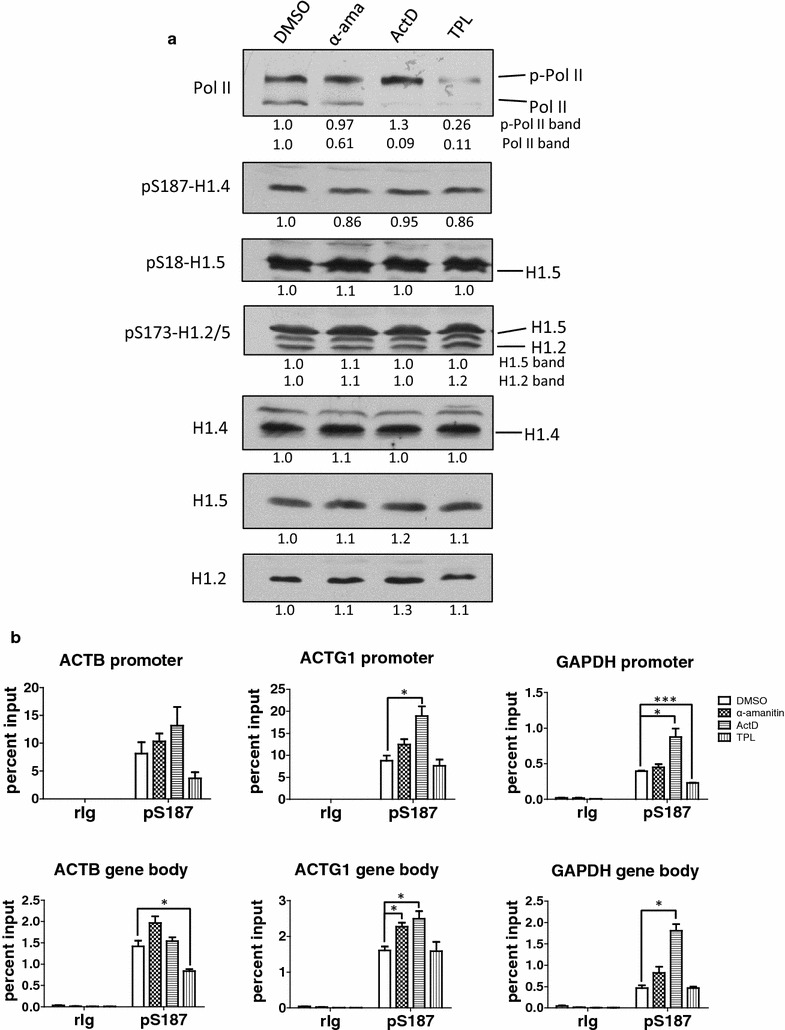
pS187-H1.4 levels are independent of RNAP II progression. **a** HeLa cells were treated with DMSO (solvent control), 50 μM α-amanitin, 500 nM actinomycin D or 200 nM triptolide for 1 h. The abundance and phosphorylation of RNA polymerase II, H1.2, H1.4 and H1.5 were analyzed by immunoblotting whole-cell lysates with the indicated antisera. The *numbers below each panel* indicate densitometry for the treated samples relative to the DMSO control sample. **b** The levels of pS187-H1.4 at the promoters and gene bodies of housekeeping genes in HeLa cells treated with DMSO (solvent control), 50 μM α-amanitin, 500 nM actinomycin D or 200 nM triptolide for 1 h were assessed by ChIP-qPCR. Negative control ChIP assays employed non-immune rabbit immunoglobulin (rIg) in place of primary antisera. The data are expressed as percent relative to input DNA (mean ± s.e.m., *p < 0.05; **p < 0.01; ***p < 0.001)

Our data on gene-specific pS187-H1.4 dynamics during NT2 cell differentiation (Fig. 2), following short-term inhibition of CDK7 or CDK9 (Fig. 4), and following CDK9 depletion (Fig. 5), suggest that H1.4-S187 phosphorylation by CDK9 could be involved in mechanisms that facilitate transcription by promoting initiation, elongation, or both processes. However, the possibility remains that enrichment of pS187-H1.4 at active genes is mediated by RNAP II-associated kinases other than CDK7 or CDK9. Consequently, we assessed how inhibiting transcription with drugs that are not kinase inhibitors affected the global and gene-specific levels of pS187-H1.4 (Fig. 6). Brief (1 h) exposure of HeLa cells to α-amanitin (50 µM), actinomycin D (500 nM) or triptolide (200 nM) elicited small or no changes in the expression of H1.2, H1.4 and H1.5, and the global levels pS18-H1.5, pS173-H1.2, pS173-H1.5 and pS187-H1.4 (Fig. 6a). Actinomycin D induced global RNAP II CTD hyperphosphorylation, consistent with prior evidence that actinomycin D and other treatments that promote P-TEFb release from 7SK snRNP complexes enhance the accumulation of CTD-hyperphosphorylated RNAP II [61, 62]. Triptolide dramatically reduced the levels of both phosphorylated and non-phosphorylated RNAP II, as expected from prior evidence that TPL inhibits the helicase activity of TFIIH by covalently binding the XPB subunit and rapidly induces proteasome-dependent degradation of the large subunit (RPB1) of RNAPII [63]. α-Amanitin reduced the level of non-phosphorylated RNAP II, but not the hyperphosphorylated forms, consistent with prior evidence that α-amanitin induces hyperphosphorylation of RNAP II CTD and RPB1 degradation [61, 64].

Remarkably, actinomycin D treatment led to a significant accumulation of pS187-H1.4 at the promoters and the bodies of ACTG1 and GAPDH genes compared to the control sample (Fig. 6b). α-Amanitin increased the level of pS187-H1.4 slightly at all of the gene regions assessed compared to control cells, but these differences were statistically significant only for the body of the ACTG1 gene. In contrast, TPL caused significant decreases in the level of pS187-H1.4 at the GAPDH promoter and the body of the ACTB gene. Taken together, the data in Fig. 6 suggest that the global and genic levels of pS187-H1.4 are not dependent on RNAP II progression, arguing against the possibility that interphase H1.4-S187 phosphorylation is mediated predominantly by kinases other than CDK9 (or possibly CDK7).

### pS187-H1.4 is co-enriched with CDK9 at specific gene loci

**Fig. 7:**
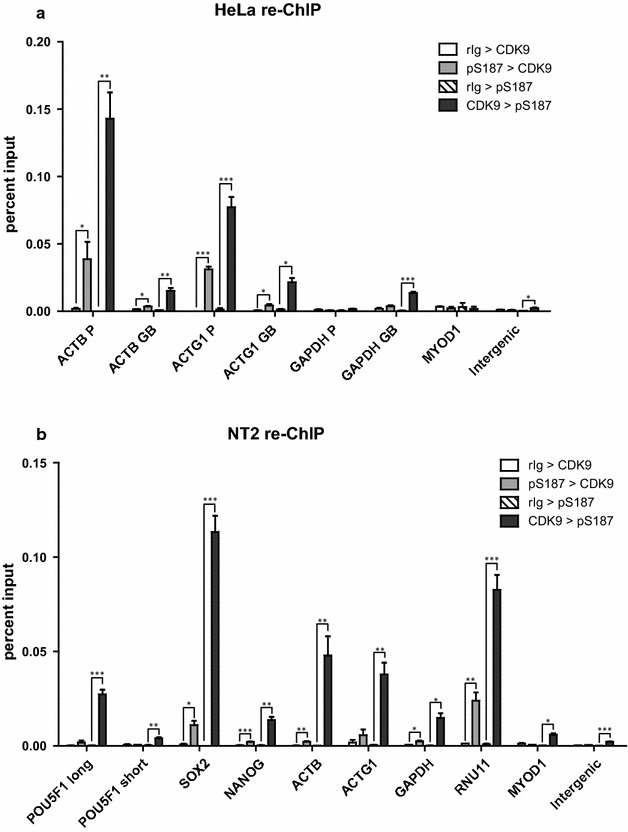
Co-enrichment of CDK9 and pS187-H1.4 at selected genes. **a** re-ChIP assays were performed on asynchronous HeLa cells using CDK9 antibody in the first ChIP followed by pS187-H1.4 antiserum in the second ChIP or vice versa. The co-enrichment of CDK9 and pS187-H1.4 at selected promoters and bodies of housekeeping genes was assessed by qPCR. **b** re-ChIP assays were performed on pluripotent NT2 cells as in (**a**). The co-enrichment of CDK9 and pS187-H1.4 at the promoters of pluripotency genes and housekeeping genes was assessed by qPCR. Negative control re-ChIP assays employed non-immune rabbit IgG (rIg) in place of primary antisera for the first ChIP. The data are expressed as percent relative to input DNA (mean ± s.e.m., *p < 0.05; **p < 0.01; ***p < 0.001)

The dependency of global and genic pS187-H1.4 levels on CDK9 expression (Fig. 5) and the enhanced enrichment of pS187-H1.4 at housekeeping genes induced by actinomycin D (Fig. 6) both suggest that the levels of pS187-H1.4 at genomic loci depend on the extent of CDK9 recruitment to those loci. We used sequential or “re-ChIP” analyses to test this hypothesis and discovered that pS187-H1.4 and CDK9 were co-enriched to a much greater extent on active housekeeping gene chromatin fragments in HeLa cells compared to fragments derived from the intergenic negative control region or the inactive MYOD1 promoter (Fig. 7a). Similarly, pS187-H1.4 and CDK9 were extensively co-enriched on active pluripotency factor and housekeeping gene chromatin fragments compared to the intergenic region or the inactive MYOD1 promoter in NT2 cells (Fig. 7b).

**Fig. 8:**
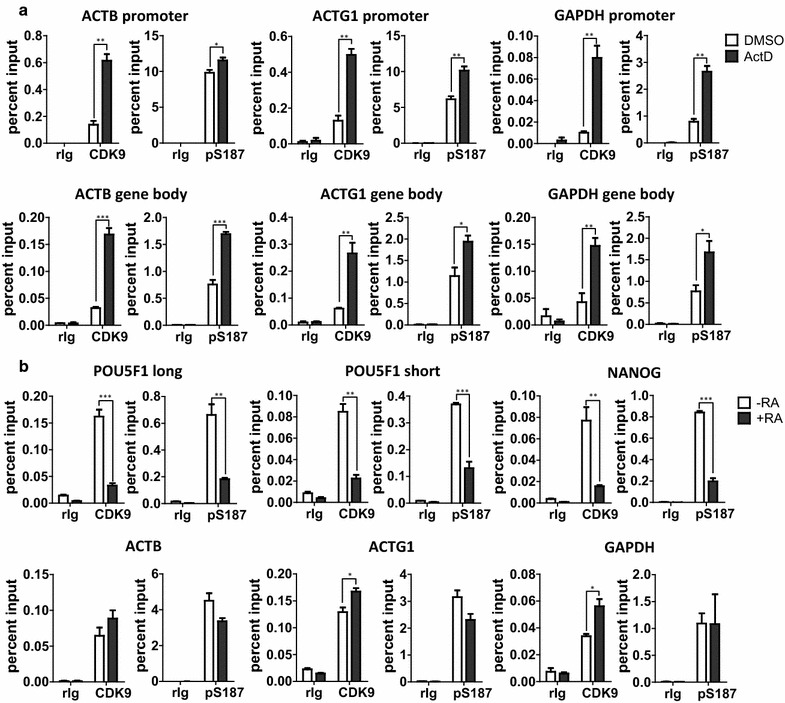
Correspondence of CDK9 and pS187-H1.4 levels at selected genes. **a** HeLa cells were treated with DMSO (solvent control) or 500 nM actinomycin D for 1 h. The levels of CDK9 and pS187-H1.4 at the promoters and gene bodies of housekeeping genes were assessed by ChIP-qPCR. **b** The levels of CDK9 and pS187-H1.4 at the transcription start sites of pluripotency factor genes and housekeeping genes in NT2 cells before and after 7 days of RA treatment were assessed by ChIP-qPCR. Negative control ChIP assays employed non-immune rabbit immunoglobulin (rIg) in place of primary antisera. The data are expressed as percent relative to input DNA (mean ± s.e.m., *p < 0.05; **p < 0.01; ***p < 0.001)

We also assessed how treatments that alter the chromatin distribution of CDK9 affected the enrichment of pS187-H1.4 at genes. Actinomycin D rapidly induced the accumulation of CDK9 at both the promoters and bodies of the ACTB, ACTG1 and GAPDH genes in HeLa cells (Fig. 8a), and this was accompanied by corresponding increases in the level of pS187-H1.4 in every case. Similarly, differentiation of NT2 cells with RA led to marked reductions in the association of CDK9 with the Oct-4 and Nanog promoters which correlated with significant decreases in the level of pS187-H1.4 at these same sites (Fig. 8b). In contrast, CDK9 binding in differentiated cells was increased at the ACTG1 and GAPDH promoters, and to a lesser extent at the ACTB promoter, and this was accompanied by non-significant reductions in the association of pS187-H1.4. Taken together, the data from our re-ChIP analyses (Fig. 7), the effect of ActD on CDK9 and pS187-H1.4 levels at housekeeping genes in HeLa cells (Fig. 8a), and the correspondence of CDK9 and pS187-H1.4 levels at pluripotency factor promoters in pluripotent and differentiated NT2 cells (Fig. 8b), suggest that CDK9 mediates H1.4-S187 phosphorylation in vivo and that the levels of pS187-H1.4 at loci reflect the extent of CDK9 recruitment to those sites. However, this simple relationship was not evident when housekeeping genes in pluripotent and differentiated NT2 cells were compared (Fig. 8b). Potential explanations for this difference include the possibility that appreciable amounts of inactive CDK9 (e.g., chromatin–associated 7SK complexes) contribute to the ChIP signal for housekeeping gene promoters in differentiated NT2 cells, whereas active P-TEFb predominates in ActD-treated cells due to its release from 7SK complexes or that pS187-H1.4 dephosphorylation is regulated differently in these distinct cellular contexts. Nonetheless, the data in Fig. 8b provide striking evidence for differences in the mechanisms that regulate the levels of CDK9 and pS187-H1.4 at specific genes.

## Discussion

### Cell differentiation has site-specific effects on H1 variant interphase phosphorylation

Multiple differences in epigenomic landscape and a more open chromatin structure distinguish pluripotent cells from their differentiated counterparts [65–67]. Differentiated cells have increased heterochromatin content over ES cells [68–70], consistent with evidence that repressive histone marks like H3K9me3 and H3K27me3 are distributed more widely in differentiated cells compared to stem cells [71]. Conversely, H3 and H4 acetylation are more prevalent in pluripotent ES cells than differentiated cells [67], and H3K9ac is enriched at a greater fraction of promoters in stem cells compared to differentiated cells [72]. The global abundance of multi-acetylated H4 diminishes during ES cell differentiation, with ES cell-specific and embryoid body-specific peaks of multi-acetylated H4 present at genes that are differentially expressed in these cell types [73]. The work presented here provides novel evidence that changes in the abundance and distribution of interphase phosphorylated forms of H1 variants are also involved in the changes in nuclear structure and gene activity that accompany cell differentiation. Phosphorylation decreases the affinity of H1-GFP fusions for chromatin binding and enhances their mobility in vivo [9]. Thus, the higher global levels of phosphorylation that we show are present at several interphase sites in pluripotent NT2 and mES cells may contribute to the weaker chromatin binding of endogenous H1 variants and the hyperdynamic mobility of H1-GFP fusions in ES cells compared to differentiated cells [74].

Significant changes in cell cycle regulation occur during pluripotent cell differentiation. Whereas S phase cells are predominant in asynchronous cultures of pluripotent NT2 cells, this fraction decreases and the proportion of G1 phase cells increases following differentiation (data not shown) [75, 76]. The levels of interphase H1 phosphorylation are expected to decrease globally with differentiation since analyses of crude H1 (i.e., the sum of the H1 variants expressed) have revealed that phosphorylation increases progressively over interphase before reaching maximum levels at mitosis [14–19]. However, it is unclear whether the progressive increase in H1 phosphorylation during interphase detected in earlier work was due to increasing levels of phosphorylation at a fixed number of sites, increased numbers of sites becoming phosphorylated, or a combination of these alternatives. Recent evidence supports the former possibility, suggesting that the majority of sites of interphase phosphorylation of H1 variants are established during G1 phase. A clear increase in the levels of phosphorylation at all interphase sites of H1.2, H1.3, H1.4 and H1.5 was observed during S phase, and this pattern was largely preserved in G2/M cells with additional hyperphosphorylated forms being identified [77]. These findings suggest that the increased abundance of G1 cells after differentiation should lead to uniform reductions in phosphorylation across interphase sites unless they are regulated in a variant-specific or site-specific fashion. Our findings that H1.2, H1.4 and H1.5 are more highly phosphorylated in asynchronous cultures of undifferentiated NT2 cells than they are after differentiation is consistent with the presence of a higher proportion of S phase cells. However, the preferential loss of pS18-H1.5 observed during NT2 cell differentiation, and the preferential loss of pS173-H1.2/5 combined with elevated pS187-H1.4 observed during mESC differentiation argue that interphase phosphorylation levels are not uniformly reduced across the distinct types of sites present in different H1 variants during cell differentiation as a consequence of the shift in cell cycle distribution. Although little is known about the numbers and nature of the pathways that regulate interphase H1 phosphorylation, our findings demonstrate that they act in conjunction with changes in the differential expression of H1 variants to regulate the phosphorylation levels on individual H1 variants in a site-specific fashion.

### Cell differentiation affects the genomic distributions of interphase phosphorylated H1 variants

Embryonic stem cells are pluripotent cells derived from the inner cell mass of the early embryo that are able to self-renew and differentiate into all three germ layers [78, 79]. A regulatory network involving transcription factors such as OCT4, SOX2 and NANOG is essential for the maintenance of pluripotency in ES cells [79, 80]. The binding of RA to RAR/RXR heterodimers at enhancers containing retinoic acid response elements (RARE) activates the transcription of RA-regulated primary response genes, including transcription factors that activate or repress their target genes [81, 82]. RA-induced repression of pluripotency factor genes is not mediated directly by RAR/RXR. Increased expression of the orphan nuclear receptor GCNF induced by RA represses OCT4 expression by promoting GCNF binding to the OCT4 promoter [83, 84]. The binding of GCNF recruits the DNA methyltransferase DNMT3 to the OCT4 promoter, facilitating its methylation and transcriptional repression. H3K9/K14 acetylation and H3K4 methylation at the OCT4 promoter decreases during differentiation, while H3K9 and H3K27 methylation levels remain constant [85]. Our data indicate that a loss of pS187-H1.4 enrichment, and possibly pS173-H1.2/5, at pluripotency factor promoters is part of the mechanism of their repression by RA in NT2 cells. In contrast, pS18-H1.5 appears to become modestly enriched at these loci when they are repressed. These differentiation-induced changes in phosphorylated H1 variant distribution appear to be gene-specific since significant changes were not detected for pS187-H1.4, pS173-H1.2/5 or pS18-H1.5 at several housekeeping genes. The elevated expression of H1.0 after cell differentiation may also facilitate the repression of genes important for self-renewal since ChIP analyses suggest that the association of HA-tagged H1.0 with pluripotency genes is enhanced in differentiated cells compared to pluripotent cells [34]. It may be significant in this regard that H1.0 lacks predicted sites for interphase phosphorylation [13].

### Regulation of interphase H1 phosphorylation

Our data imply that enrichment of pS187-H1.4 at the promoters of pluripotency factor genes in pluripotent cells, and at the promoters of housekeeping genes (and presumably other types of genes) in both pluripotent and differentiated cells, is associated with their transcriptional activity. Interphase H1 phosphorylation has been implicated in facilitating transcription in a gene-specific fashion in several model systems. ChIP analyses with an antisera that recognizes multiple H1 phosphorylations suggest that H1 phosphorylation levels correlate directly with the hormone-inducibility and transcriptional competency of the murine mammary tumor virus (MMTV) promoter [29, 86]. This is consistent with subsequent work showing that pS187-H1.4 is enriched at hormone-response elements shortly after hormone stimulation, suggesting that this pS187-H1.4 enrichment facilitates the transcription of their target genes [23]. However, little is known about the mechanisms responsible for the targeted enrichment of pS187-H1.4 or other interphase phosphorylated forms of H1.

CDK2 has been suggested to be an interphase H1 kinase, but the evidence is equivocal. FRAP analyses suggested that the mobility of GFP-H1.4, but not GFP-M1-5 (all five S/TPXZ sites in H1.4 mutated to APXZ), was positively correlated with the level of CDK2 activity, and this difference was minimized upon CDK2 inhibition [30]. However, later work showed that three out of the five CDK motifs investigated are TPXZ motifs that are phosphorylated exclusively during mitosis [23]. Inhibitors thought to be selective for CDK2 such as CVT-313 and roscovitine decreased global levels of phosphorylated H1 and its association with the MMTV promoter [29], and roscovitine and olomoucine diminished H1 phosphorylation at replication foci [87], but several issues affect these studies. Both employed antisera raised against phosphorylated *Tetrahymena* macronuclear H1, which lacks SPXZ motifs [88], to detect phosphorylated H1 even though the sites in metazoan H1 variants that are recognized by this antisera have not been established. Moreover, CVT-313, roscovitine and olomoucine are now known to inhibit additional CDKs with similar potency [89–92], and prolonged treatments with these drugs can arrest cell cycle progression [91, 93] and consequently affect H1 phosphorylation levels indirectly. Here, we used a time course experiment to show that treatments as long as 24 h with NU6140, an inhibitor with greater selectivity for CDK2 [44], did not reduce the global level of pS187-H1.4 or pS18-H1.5 in NT2 cells, providing strong evidence that CDK2 is not involved in regulating phosphorylation of H1 at these two sites.

Multiple lines of evidence presented here strongly implicate CDK9 as the predominant interphase kinase for H1.4-S187 in NT2 and HeLa cells. Brief (one hour or less) treatments with the preferential CDK9 inhibitor FLVP significantly diminish the global level of pS187-H1.4 in both NT2 cells (Fig. 3) and HeLa cells (Fig. 4), without diminishing the levels of pS18-H1.5 or pS173-H1.2/5 (Fig. 4). Short FLVP treatment essentially abolished pS187-H1.4 enrichment at housekeeping genes in HeLa cells (Fig. 4). Depletion of CDK9 in HeLa cells significantly decreased global H1.4-S187 phosphorylation and the association of pS187-H1.4 with housekeeping genes, while the global levels of pS18-H1.5 and pS173-H1.2/5 were slightly increased (Fig. 5). Moreover, re-ChIP analyses show that CDK9 and pS187-H1.4 are specifically co-enriched on housekeeping gene chromatin fragments recovered from HeLa and NT2 cells and also on pluripotency factor chromatin fragments recovered from NT2 cells (Fig. 7). Taken together, these findings provide strong evidence that CDK9 mediates H1.4-S187 phosphorylation which is consistent with prior evidence that H1 interacts with P-TEFb in vivo [31]. Remarkably, our findings also imply that the H1.5-S18 and H1.2/5-S173 interphase phosphorylations are mediated by distinct kinases. However, our data do not exclude the possibility that H1.4-S187 may be targeted by additional interphase kinases. Brief, highly selective inhibition of CDK7 by THZ1 also decreased the levels of pS187-H1.4 globally and at specific gene loci (Fig. 4), but further work is required to determine the extent to which this reflects the possible direct phosphorylation of H1.4-S187 or is secondary to diminished activation of CDK9 [57]. Our data are consistent with a role for CDK7 via either mechanism.

Given the roles of CDK7 and CDK9 in regulating RNAP II transcription [47, 57], the formal possibility remains that the effects of CDK7 and CDK9 inhibition on H1 phosphorylation are secondary to transcription deficits elicited by these drugs. We suggest that this is unlikely since we limited inhibitor treatments to just one hour. Furthermore, our findings that inhibition of RNAP II transcription with α-amanitin, actinomycin D or triptolide had relatively minor effects on global pS187-H1.4 levels (Fig. 6) compared to kinase inhibition by THZ1 and FLVP (Fig. 4) also argue against this possibility and suggest that H1.4-S187 phosphorylation does not depend directly on RNAP II progression. In fact, the enhancements in global RNAP II CTD phosphorylation and the association of pS187-H1.4 at gene promoters and bodies elicited by brief treatment with ActD (Fig. 6) provide further evidence that CDK9 is a *bona fide* interphase kinase for H1.4-S187 since ActD induces the release of active P-TEFb from 7SK snRNA complexes [61, 94, 95]. Moreover, we show that CDK9 and pS187-H1.4 are co-enriched at housekeeping gene promoters and gene bodies in ActD-treated HeLa cells but are co-depleted at pluripotency factor gene promoters after NT2 cells differentiate (Fig. 8).

Our collective data suggest that recruitment of existing active P-TEFb complexes, or P-TEFb released from 7SK complexes, to sites of transcription results in the accumulation and enrichment of pS187-H1.4 that facilitates RNAP II transcription by promoting the transient dissociation of H1.4 or possibly by other mechanisms. A variety of mechanisms have been implicated in releasing P-TEFb from 7SK complexes, including post-translational modification of 7SK snRNP components and direct interactions with RNA binding proteins or transcriptional regulators [96, 97]. The RNA splicing factor SRSF2 (also known as SC-35) was found to be part of the 7SK complex assembled at gene promoters. The binding of SRSF2 to promoter-associated nascent RNA triggers the coordinated release of SRSF2 and P-TEFb from the 7SK complex, reminiscent of the mechanism used by HIV Tat/TAR to activate the transcription of HIV genes [98]. The promoter-bound DEAD-box RNA helicase DDX21 was found to be recruited to promoters of RNAP II-transcribed genes encoding ribosomal proteins and snoRNAs and promote their activation by releasing P-TEFb from the 7SK snRNP in a helicase-dependent manner [99]. These different mechanisms for releasing P-TEFb from 7SK complexes share the common feature that the activation of P-TEFb occurs on chromatin where transcription and pre-mRNA processing occurs. Our findings suggest that H1.4-S187 phosphorylation at gene promoters/TSSs occurring conjointly with phosphorylation of the RNAP II-CTD, DSIF and NELF by P-TEFb may facilitate transcriptional elongation by promoting the release of promoter-proximal paused RNAP II, but further work is required to distinguish between this and other possible consequences of H1.4-S187 phosphorylation.

Recent data suggest a possible mechanism for our observations that the levels of CDK9 and pS187-H1.4 at pluripotency factor gene promoters are reduced by differentiation (Figs. 2, 8b). BRD4 and acetylated histone H4 are enriched at the OCT4 enhancer and promoter regions in ESCs, and their enrichment decreases upon differentiation and embryoid body formation [73]. BRD4 binding to acetylated H4 mediates CDK9 recruitment at pluripotency factor genes such as OCT4 that are associated with super-enhancers in ESCs. Inhibiting chromatin binding by BRD4 with a specific bromodomain inhibitor impaired CDK9 recruitment at the OCT4 enhancer and promoter regions and reduced the expression of pluripotency genes [100]. These findings suggest that reduced BRD4 enrichment at OCT4 and other pluripotency genes leads to less CDK9 recruitment and consequently diminished H1.4-S187 phosphorylation following cell differentiation.

### Functional diversity of H1 variants

An obvious difference that distinguishes the amino acid sequences of metazoan H1 variants from each other is the number and positions of SPXZ sites they possess for interphase phosphorylation. Human H1.1 and H1.3 each have one at the same relative position (S183 or S189). H1.2 also has one, but at a different position (S173). H1.4 has two (S172 and S187) and H1.5 has three (S18, S173 and S189). Other human H1 variants either lack SPXZ sites or they occur at non-conserved positions [13]. These differences in SPXZ motif conservation may enable phosphorylation to impart different functions to individual H1 variants by altering their conformation, DNA binding and other properties in specific ways [101]. Our evidence that pS187-H1.4, but not pS18-H1.5, is preferentially associated with active genes (vide infra) [23, 24] is consistent with this possibility. The differences we observed in the regulation of phosphorylation at H1.4-S187 versus H1.2-S173, H1.5-S173 and H1.5-S18 also support this hypothesis. The involvement of CDK7 and CDK9 revealed here in regulating H1.4-S187 phosphorylation may contribute to the unique requirement of H1.4 among H1.0 and H1.1-H1.5 for the viability of human breast cancer cells [102]. Moreover, these differences in phosphorylation regulation, together with the changes we observed for interphase phosphorylation of H1.2, H1.4 and H1.5 during pluripotent cell differentiation, suggest that these pathways are likely to play distinct roles in developmental regulation of gene expression.

## Conclusions

In this study, we demonstrated that interphase phosphorylation of H1 variants is reduced preferentially at specific sites during pluripotent cell differentiation and that the enrichment of pS187-H1.4, but not pS18-H1.5, is positively correlated with transcription. Brief inhibition of CDK9 or CDK7 with specific inhibitors, or depletion of CDK9, decreased global and gene-specific levels of pS187-H1.4. Remarkably, inhibiting RNAP II transcription with actinomycin D significantly enhanced co-enrichment of pS187-H1.4 and CDK9 at housekeeping genes. In contrast, the global levels of pS18-H1.5 or pS173-H1.2/5 are not affected by any of the above treatments, suggesting that these interphase phosphorylations are regulated by different mechanisms. Our findings suggest that H1.4-S187 is a *bona fide* substrate for CDK9 that is phosphorylated during transcriptional activation, and that reductions in the levels of CDK9 and pS187-H1.4 at pluripotency factor gene promoters contribute to their repression of during cell differentiation. Interphase phosphorylation at other sites in H1 variants appears to be regulated by different kinase pathways, and this is expected to contribute to functional diversity among different H1 variants.

## Additional files

**Additional file 1: Table S1.** List of ChIP-qPCR primers.

**Additional file 2: Figure S1.** Validations of custom H1 antibodies that have not been published previously. *A*, Recombinant H1 variants were analyzed by immunoblotting with our custom antisera for H1.5 and H1.0. *B*, PCA-extracted crude H1 from WI-38 VA-13 cells and a mixture of recombinant H1.5 and H1.0 were analyzed by immunoblotting with our custom antisera against pS18-H1.5. *C*, Antisera against pS18-H1.5 was mock-treated (none) or preadsorbed with pS18-H1.5 antigen peptide (pS18) or the corresponding non-phosphorylated peptide (S18) prior to immunoblotting with HeLa whole-cell lysate. *D*, HeLa and WI-38 VA-13 whole-cell lysates (WCL) and PCA-extracted crude H1 (PCAS) from WI-38 VA-13 cells were analyzed by immunoblotting with our custom antisera against H1.5 and H1.0.

**Additional file 3: Figure S2.** H1 variant expression and phosphorylation in differentiating NT2 cells. Crude H1 in acid extracts of nuclei isolated from NT2 cells induced to differentiate with 10 μM retinoic acid for 0, 1, 3 or 7 days was fractionated by hydrophobic interaction chromatography (HIC). Eluate absorbance at 214 nm (*Y* axis) is plotted relative to time (*X* axis) for equivalent portions of each separation. The relative elution positions of H1.2, H1.3, H1.4 and H1.5 and the phosphorylation stoichiometry of their major interphase forms, as characterized previously [23], are indicated above the 0 day trace. H1.0 coelutes as a broad peak that overlaps with both phosphorylated and non-phosphorylated H1.5 (data not shown).

**Additional file 4: Figure S3.** pS187-H1.4 is preferentially enriched in active chromatin. *A*, HeLa cells were treated with DMSO or 500 nM ActD for 1 h and the levels of CDK9 and pS187-H1.4 at the promoters of ACTG1, GAPDH or MYOD1 and one intergenic region were assessed by ChIP-qPCR. *B*, HeLa cells were treated with DMSO or 1 µM FLVP for 1 h and the levels of CDK9 and pS187-H1.4 at the promoters of ACTG1, GAPDH or MYOD1 and one intergenic region were assessed by ChIP-qPCR. Negative control ChIP assays employed non-immune rabbit IgG (rIg) in place of primary antisera for the first ChIP. The data are expressed as percent relative to input DNA (mean ± s.e.m., *: p < 0.05, **: p < 0.01, ***: p < 0.001).

## Abbreviations

ActD: actinomycin D
CAK: CDK-activating kinase
CDK: cyclin-dependent kinase
ChIP: chromatin immunoprecipitation
CTD: C-terminal domain
ESC: embryonic stem cell
FLVP: flavopiridol
LIF: leukemia inhibitory factor
P-TEFb: positive transcription elongation factor b
RA: retinoic acid
TPL: triptolide
TSS: transcription start site
