# Supplementary material for: Site-specific regulation of histone H1 phosphorylation in pluripotent cell differentiation
Source: Epigenetics Chromatin. 2017 May 22;10:29. doi: 10.1186/s13072-017-0135-3 (PMC5440973; doi:10.1186/s13072-017-0135-3)
Supplement: Supplementary file 1 — Additional file 1: Table S1. List of ChIP-qPCR primers. [file 13072_2017_135_MOESM1_ESM.docx]

Additional file 1: Table S1. Primers used for ChIP-qPCR

| POU5F1 long forward | CTTCGCAAGCCCTCATTT |
| --- | --- |
| POU5F1 long reverse | AGGTCCGAGGATCAACCC |
| POU5F1 short forward | ATGCCATGTTATAGTTTGTG |
| POU5F1 short reverse | GCTGCTAAGTTCTGGGTTA |
| SOX2 forward | CGCTAGAAACCCATTTATTCC |
| SOX2 reverse | CCTAGTCTTAAAGAGGCAGCAA |
| NANOG forward | TATTATGCAGGCAACTCA |
| NANOG reverse | AGTATAGAGGAAGAGGAGGA |
| ACTB promoter forward | GAAAGTTGCCTTTTATGGCTCG |
| ACTB promoter reverse | TTACCTGGCGGCGGGTGT |
| ACTG1 promoter forward | CGGCTTTCGGAAAGATCG |
| ACTG1 promoter reverse | GAGCGGCGGAAGAACAGA |
| GAPDH promoter forward | TTGGGCTGGGACTGGCTGAG |
| GAPDH promoter reverse | GGCTGACTGTCGAACAGGAGG |
| RNU11 forward | ACACGTAGGGCAACTCGA |
| RNU11 reverse | AAGCACCACTTACTCCAAA |
| ACTB gene body forward | CGGGAAATCGTGCGTGAC |
| ACTB gene body reverse | GGAAGGAAGGCTGGAAGAGTG |
| ACTG1 gene body forward | CCATCGTCCACCGCAAAT |
| ACTG1 gene body reverse | TCGAAGGCTTATTCCAGTTTC |
| GAPDH gene body forward | CTGCTGTAGGCTCATTTGCA |
| GAPDH gene body reverse | GAGGCTGTTGTCATACTTCTCA |
| MYOD1 forward | GCCACAACGGACGACTTCTATG |
| MYOD1 reverse | CGAGTGCTCTTCGGGTTTCAG |
| Intergenic forward | ATTTGAGGGCAGGAAGC |
| Intergenic reverse | GCGCAGCTAGAATAAAGCA |
