# Supplementary figures and images for: Site-specific regulation of histone H1 phosphorylation in pluripotent cell differentiation
Source: Epigenetics Chromatin. 2017 May 22;10:29. doi: 10.1186/s13072-017-0135-3 (PMC5440973; doi:10.1186/s13072-017-0135-3)

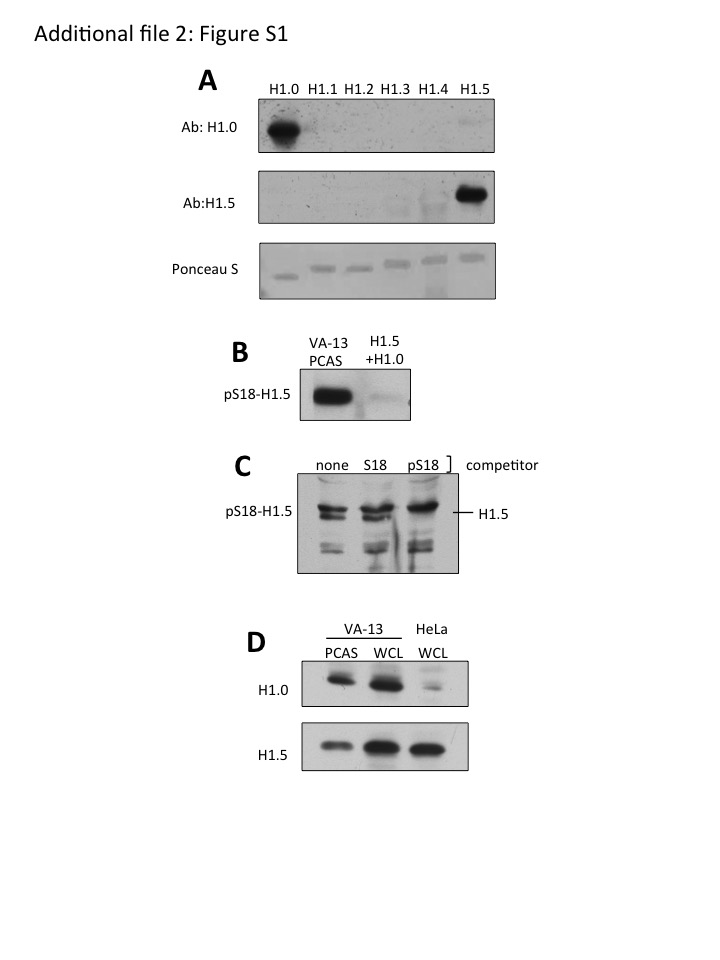

Supplement: Supplementary file 2 — Additional file 2: Figure S1. Validations of custom H1 antibodies that have not been published previously. A, Recombinant H1 variants were analyzed by immunoblotting with our custom antisera for H1.5 and H1.0. B, PCA-extracted crude H1 from WI-38 VA-13 cells and a mixture of recombinant H1.5 and H1.0 were analyzed by immunoblotting with our custom antisera against pS18-H1.5. C, Antisera against pS18-H1.5 was mock-treated (none) or preadsorbed with pS18-H1.5 antigen peptide (pS18) or the corresponding non-phosphorylated peptide (S18) prior to immunoblotting with HeLa whole-cell lysate. D, HeLa and WI-38 VA-13 whole-cell lysates (WCL) and PCA-extracted crude H1 (PCAS) from WI-38 VA-13 cells were analyzed by immunoblotting with our custom antisera against H1.5 and H1.0. [file 13072_2017_135_MOESM2_ESM.jpg]

## Slide 1
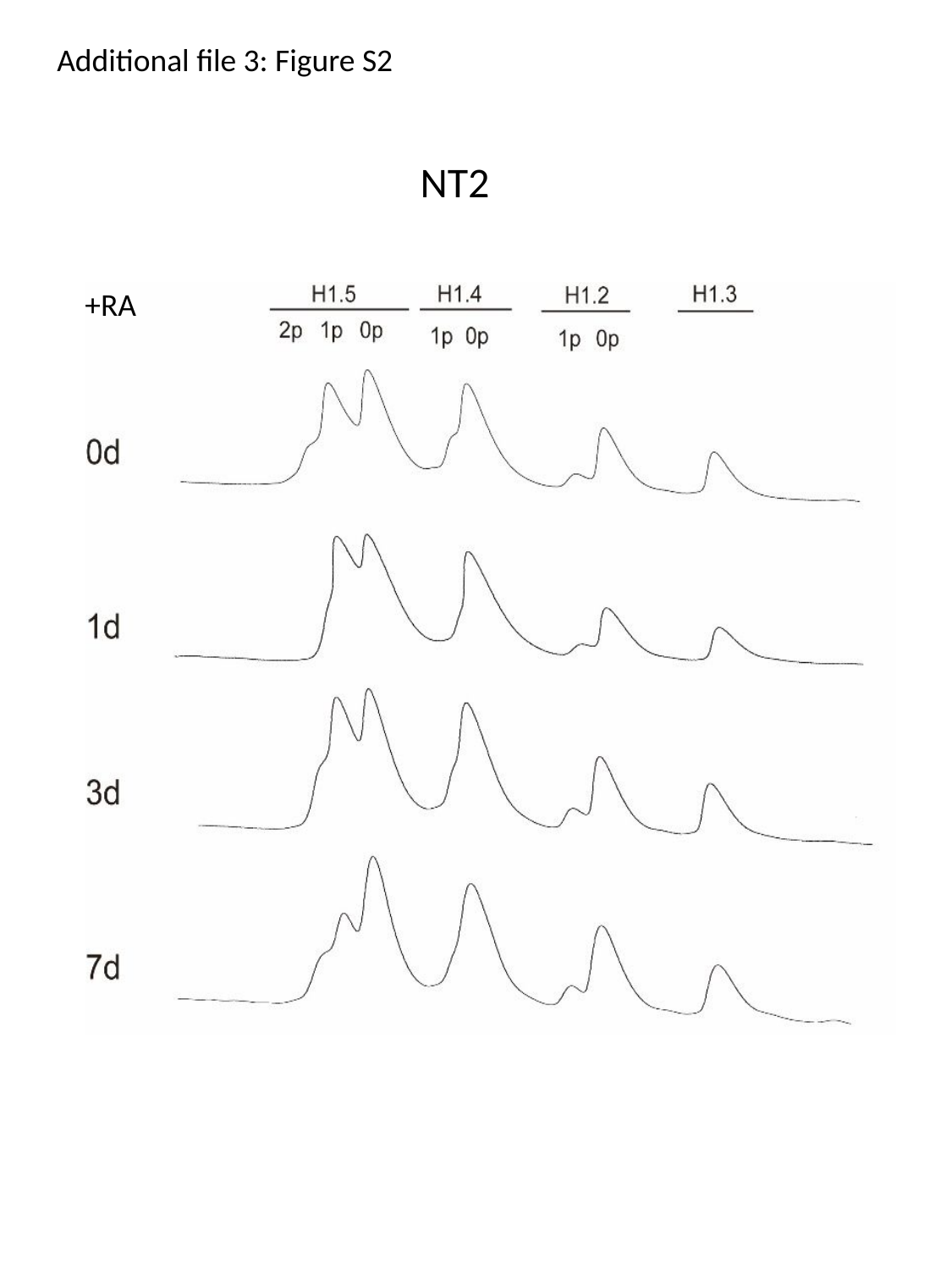

Additional file 3: Figure S2
NT2
+RA

Supplement: Supplementary file 3 — Additional file 3: Figure S2. H1 variant expression and phosphorylation in differentiating NT2 cells. Crude H1 in acid extracts of nuclei isolated from NT2 cells induced to differentiate with 10 μM retinoic acid for 0, 1, 3 or 7 days was fractionated by hydrophobic interaction chromatography (HIC). Eluate absorbance at 214 nm (Y axis) is plotted relative to time (X axis) for equivalent portions of each separation. The relative elution positions of H1.2, H1.3, H1.4 and H1.5 and the phosphorylation stoichiometry of their major interphase forms, as characterized previously [23], are indicated above the 0 day trace. H1.0 coelutes as a broad peak that overlaps with both phosphorylated and non-phosphorylated H1.5 (data not shown). [file 13072_2017_135_MOESM3_ESM.pptx]

## Slide 1
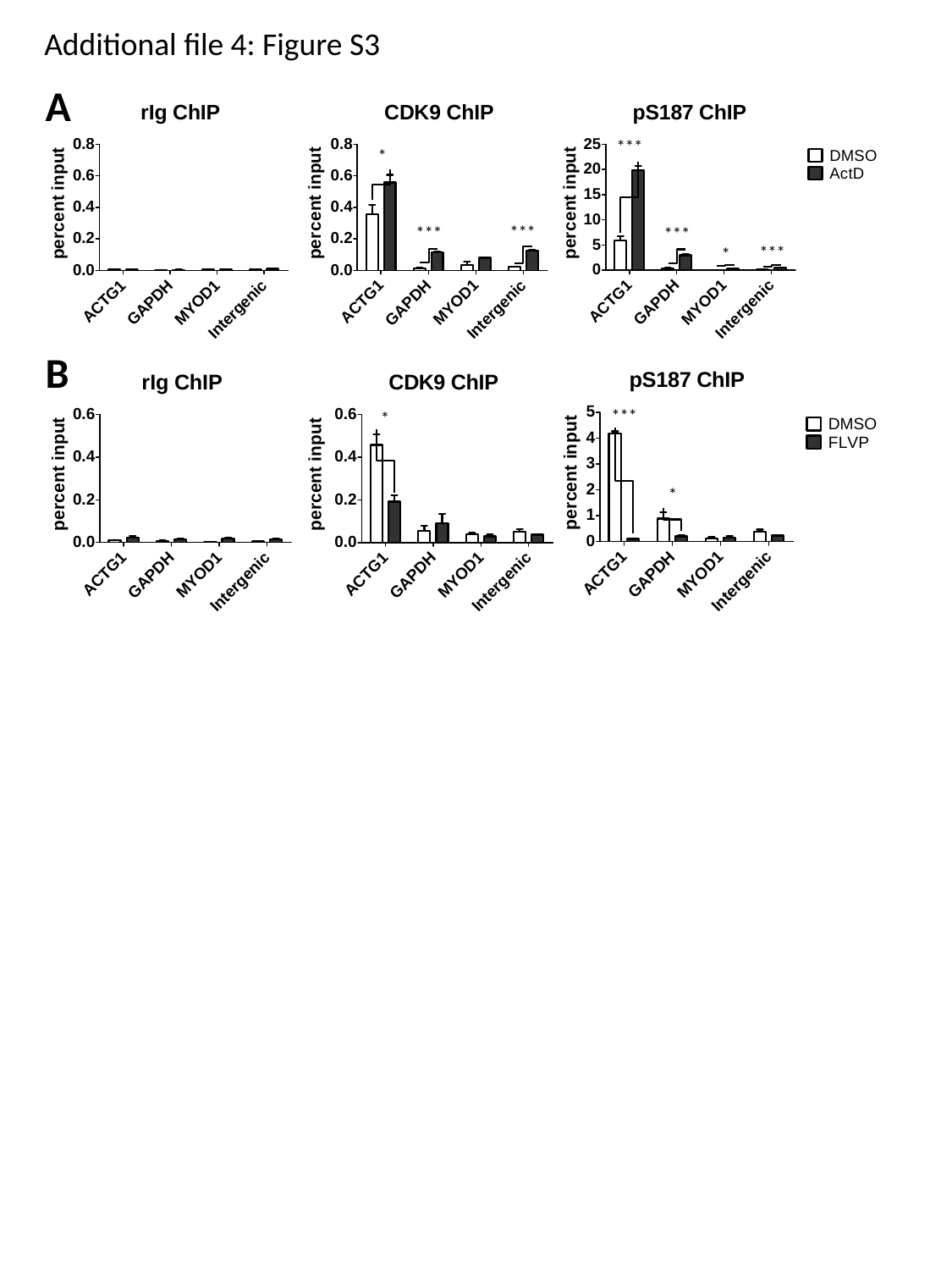

Additional file 4: Figure S3
A
***
*
***
***
***
***
*
B
***
*
*

Supplement: Supplementary file 4 — Additional file 4: Figure S3. pS187-H1.4 is preferentially enriched in active chromatin. A, HeLa cells were treated with DMSO or 500 nM ActD for 1 h and the levels of CDK9 and pS187-H1.4 at the promoters of ACTG1, GAPDH or MYOD1 and one intergenic region were assessed by ChIP-qPCR. B, HeLa cells were treated with DMSO or 1 µM FLVP for 1 h and the levels of CDK9 and pS187-H1.4 at the promoters of ACTG1, GAPDH or MYOD1 and one intergenic region were assessed by ChIP-qPCR. Negative control ChIP assays employed non-immune rabbit IgG (rIg) in place of primary antisera for the first ChIP. The data are expressed as percent relative to input DNA (mean ± s.e.m., *: p < 0.05, **: p < 0.01, ***: p < 0.001). [file 13072_2017_135_MOESM4_ESM.pptx]
